# Microfabrication and Surface Functionalization of Soda Lime Glass through Direct Laser Interference Patterning

**DOI:** 10.3390/nano11010129

**Published:** 2021-01-08

**Authors:** Marcos Soldera, Sabri Alamri, Paul Alexander Sürmann, Tim Kunze, Andrés Fabián Lasagni

**Affiliations:** 1 Institute of Manufacturing Science and Engineering, Technische Universität Dresden, George-Bähr-Str. 3c, 01069 Dresden, Germany; andres_fabian.lasagni@tu-dresden.de; 2 PROBIEN-CONICET, Universidad Nacional del Comahue, Buenos Aires 1400, Neuquén 8300, Argentina; 3 Fraunhofer Institute for Material and Beam Technology IWS, Winterbergstr. 28, 01277 Dresden, Germany; sabri.alamri@iws.fraunhofer.de (S.A.); paul.suermann@iws.fraunhofer.de (P.A.S.); kunze.tim@iws.fraunhofer.de (T.K.)

**Keywords:** glass micro-structuring, direct laser interference patterning, laser-induced periodic surface structures, multi-photon absorption, wettability, diffraction gratings

## Abstract

All-purpose glasses are common in many established and emerging industries, such as microelectronics, photovoltaics, optical components, and biomedical devices due to their outstanding combination of mechanical, optical, thermal, and chemical properties. Surface functionalization through nano/micropatterning can further enhance glasses’ surface properties, expanding their applicability into new fields. Although laser structuring methods have been successfully employed on many absorbing materials, the processability of transparent materials with visible laser radiation has not been intensively studied, especially for producing structures smaller than 10 µm. Here, interference-based optical setups are used to directly pattern soda lime substrates through non-lineal absorption with ps-pulsed laser radiation in the visible spectrum. Line- and dot-like patterns are fabricated with spatial periods between 2.3 and 9.0 µm and aspect ratios up to 0.29. Furthermore, laser-induced periodic surface structures (LIPSS) with a feature size of approximately 300 nm are visible within these microstructures. The textured surfaces show significantly modified properties. Namely, the treated surfaces have an increased hydrophilic behavior, even reaching a super-hydrophilic state for some cases. In addition, the micropatterns act as relief diffraction gratings, which split incident light into diffraction modes. The process parameters were optimized to produce high-quality textures with super-hydrophilic properties and diffraction efficiencies above 30%.

## 1. Introduction

Glass offers outstanding properties for a wide range of applications where high optical transparency, chemical stability, heat resistance, hardness, or biological compatibility are needed. Moreover, in recent years, technical glasses have become an essential material for emerging markets such as biomedical devices, micro-optics and photonics, biotechnology, and microfluidics [1,2,3,4,5,6]. Functionalization of glasses via surface modification has opened an even broader scope for these materials, showing attractive features for products with a high-added value [7,8,9,10]. For instance, surfaces with tuned hydrophilicity can enhance the filling of microchannels in microfluidics chips without the need of added pumps or valves [11], whereas large area super-hydrophobic or super-hydrophilic glasses can be used in anti-fogging and self-cleaning applications [12,13]. Super-hydrophilic surfaces immersed in water can repel oil contaminants due to the differences in the surface tensions of the liquids, paving the way for novel oil/water separation applications [14,15]. In addition, such underwater self-cleaning surfaces can also prevent biofouling, which could be attractive for the food processing sector, biomedical devices, and marine industry [16]. As another example, surface relief gratings etched in glasses have shown the capability to enhance light absorption in thin film Si solar cells by diffraction and scattering, resulting in an increased power conversion efficiency up to 48% [17,18,19]. Moreover, the combination of super-hydrophilicity with high optical transparency and scattering on textured glass has shown the potential for photovoltaic modules with self-cleaning and increased energy harvesting abilities [20,21]. These examples demonstrate, among others, the endless possibilities for functionalized glass materials and their potential impact in many growing economical markets.

In order to functionalize the surface of these materials, special techniques able to induce chemistry-based and topography-based surface changes are necessary. For instance, plasma treatments have been used to tailor the wettability of glass substrates by depositing polymeric coatings with a controlled stoichiometry [22,23,24]. From the topographical point of view, chemical treatments created, in most cases, a thin coating with nano-roughness that can be controlled by adjusting the process parameters. However, these methods offer limited possibilities to fabricate deterministic surface microstructures with tunable geometrical parameters, which can be useful to induce light diffraction [25,26,27]. 

Among the numerous methods and approaches developed to fabricate deterministic microstructures on glass surfaces, those based on lithographic techniques combined with wet chemical etching or reactive ion etching are the widest spread [28,29,30,31,32]. Lithography is a well-established method in both lab-scale prototyping and industrial manufacturing able to pattern features down to the nanoscale with high aspect ratios and excellent texture uniformity. However, it is a multiple step process that requires a prefabricated mask and uses hazardous chemicals [33].

Laser-based structuring methods have appeared in recent decades as an attractive alternative to lithography-based techniques due to the continuously decreasing costs of the laser sources, combined with their increasing output power and high flexibility [34,35,36]. The laser-based micro-structuring of glass can be achieved by direct or indirect means. As an example of the latter case, the laser-induced micro-plasma ablation (LIMPA) method relies on the irradiation of a metallic absorber placed on the backside of a transparent material, e.g., fused silica. The absorbed radiation in the metal generates a plasma plume in the confined space between metal and dielectric with enough energy to ablate the materials [37,38,39,40]. In contrast, the direct laser-based processing of glasses usually requires the use of either ultraviolet laser radiation with fluences higher than 10 J/cm^2^ or laser sources providing ultra-short laser pulses (USP, in the ps or fs regime), whose high-peak intensities initiate nonlinear absorption processes [41,42,43,44,45,46]. This topic has received considerable attention in recent years and the main processes can be summarized as follows.

- Multiphoton ionization: an electron in the valence band can absorb several visible or near-infrared photons and gain enough energy to cross the band gap [47].
- Tunneling photoionization: the strong electric field suppresses the Coulomb barrier and allows the electron to tunnel through. The free electrons created by nonlinear photoionization can then absorb more energy from the laser pulse by inverse bremsstrahlung. If the energy of the free carriers becomes high enough, they can also promote an electron from the valence to the conduction band by impact ionization, leading to an avalanche process [48].

One of the most distinguishing features of two-photon absorption is that the amount of absorbed power in a thin layer of the medium is proportional to the square of the light intensity (or power), while, in one-photon absorption, the ratio of absorption depends linearly with respect to intensity [49]. Therefore, considering a Gaussian beam, the absorption rate drops quadratically, moving from the center toward the periphery of the beam and any linear variation in the power would produce a quadratic variation of the absorption coefficient and, therefore, of the ablation depth [50]. As a result, the process window for the two-photon microfabrication in non-absorbing materials is very narrow, compared with linear absorption.

Besides, the interaction of USP with the material gives rise to laser-induced periodic surface structures (LIPSS), which were recently observed in transparent ceramic materials like fused silica [51], quartz [52], sapphire [53,54], soda-lime-silicate, and borosilicate glasses [55]. Although the micro-texturing based on LIPSS is a relatively simple process, features with high aspect ratios are hard to achieve and their periodicity is mainly dictated by the used radiation wavelength and its polarization state [56].

A more flexible approach for the fabrication of periodic structures is the Direct Laser Interference Patterning (DLIP) method, by which two or more coherent beams are overlapped at the material surface to generate an interference pattern. If the local intensity is high enough to initiate the ablation, a periodic pattern can be directly engraved on the material [57]. Furthermore, controlling the number of interfering beams and their overlapping angle, the radiation polarization, and deposited fluence, as well as different textures shapes, periodicities, and aspect ratios are achievable. In addition, the material properties can also be tuned [58,59,60,61]. Particularly, using two laser beams, line-like patterns can be created, whereas overlapping four beams with the same polarization at the same incidence angle as well as a dot-like array with square symmetry can be produced. Although a few pioneering works on direct structuring by interfering ultra-short laser pulses on transparent ceramics, such as amorphous SiO_2_, sapphire, SiC, TiO_2_, diamond, among others, have been reported [62,63,64], no systematic study has been done yet to optimize the laser processing parameters and link them with controlled surface properties.

In this contribution, the processability of soda lime glass substrates using DLIP with USP and visible radiation (532 nm) is addressed. In a single DLIP step, line-like and dot-like arrays with different periodicities and aspect ratios are, thus, structured. The textured glasses showed multifunctional properties, as the surface developed a super-hydrophilic characteristic combined with the ability to diffract white light into well-defined viewing angles.

## 2. Materials and Methods 

### 2.1. Glass Substrates 

Soda lime glass sheets (100 mm × 100 mm × 5 mm) fabricated by the float-glass method (Aachener Quarzglas-Technologie Heinrich GmbH & Co. KG, Aachen, Germany) were used in all the experiments. This glass is resistant to almost all acids, salts, and their solutions and, despite its low cost, it is characterized by excellent thermal, optical, and mechanical properties, making it a commonly used all-purpose glass material. This soda lime glass has a transmission of approximately 90% at a wavelength of 532 nm, which is the wavelength of the used laser systems (as described in Section 2.2).

### 2.2. Direct Laser Interference Patterning Setups

**Figure 1:**
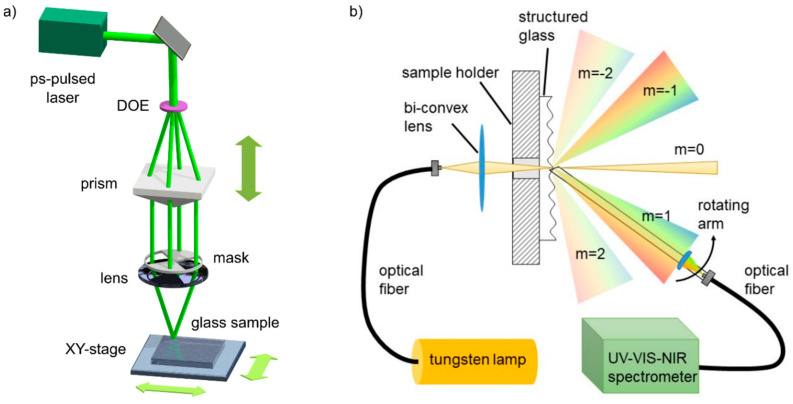
(**a**) Schematic setup of the four beams DLIP system used to structure glass. The mask is inserted to block two beams and letting the other two beams to interfere at the sample’s surface. (**b**) Characterization setup used to measure the angular and spectral intensity of the transmitted diffracted modes by the structured glass samples.

Two different DLIP systems were used to pattern either the line-like or dot-like textures. To pattern the former textures, a 12 ps pulsed laser source (EdgeWave GmbH, Würselen, Germany) with an output power of 61.4 W at a wavelength of 532 nm and at a repetition rate of 10 kHz was used. In turn, a laser source (neoLASE GmbH, Hanover, Germany) with a maximum output power of 2.7 W emitting 70 ps pulses at a wavelength of 532 nm at a repetition rate of 1 kHz was employed for structuring dot-like textures. Both DLIP systems are based on the flexible DLIP approach [65] shown schematically in Figure 1. The primary beam coming from the laser source is split into four beams by a diffractive optical element (DOE), which are then parallelized by a prism. Afterward, the secondary beams are focused by a convex lens on the sample surface. Since only two beams need to be overlapped on the sample to produce the line-like patterns, a mask is introduced before the lens to block two opposite secondary beams. By adjusting the distance between the DOE and prism, the user can easily set the overlapping angle and, thus, the texture spatial period. The samples were processed in ambient conditions at a room temperature of 22 °C and humidity of 35%.

### 2.3. Structuring Strategy

**Figure 2:**
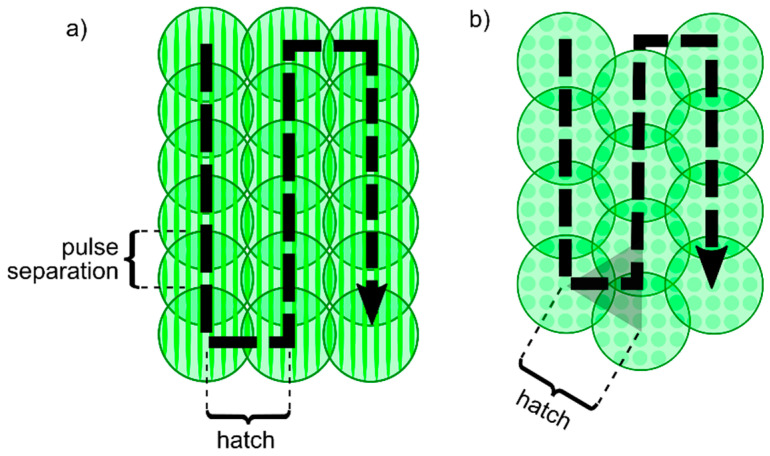
Patterning strategies followed to pattern (**a**) line-like textures with two-beam Direct Laser Interference Patterning (DLIP) and (**b**) dot-like textures with four-beam DLIP.

To pattern large areas, the followed strategies are represented in Figure 2. In the case of the two-beam setup, after firing each laser pulse, the stage where the sample is mounted moves vertically a pulse separation distance *p*_s_, which can be tuned to deposit different amounts of an accumulated fluence dose (Figure 2a). The ratio between the spot size *φ* and the pulse separation distance *p*_s_ yields the effective number of applied pulses on the same area *N*. When a vertical line is completed, the stage moves sideways along a distance called hatch, which must be a multiple of the used spatial period to avoid destroying the periodic texture.

In turn, Figure 2b shows the patterning strategy used for fabricating the dot-like textures using four-beam DLIP. In this case, the sample is placed at the specified position, then it is irradiated *N* times until it moves to the next position. A compact hexagonal array of irradiated spots was textured with the hatch distance displaced diagonally, as shown in Figure 2b. All the structured areas had a size of 5 mm × 5 mm. Table 1 lists the fixed and variable process parameters used in this work. With the used DLIP setups, the maximum patterning throughputs for the two-beam and four-beam configurations were 3.3 cm^2^/min and 2.4 cm^2^/min, respectively.

### 2.4. Topography Characterization

The fabricated DLIP structures were characterized by confocal microscopy (LeicaSCAN DCM 3D, Leica Camera, Wetzlar, Germany) with a 150X magnification objective, providing a lateral and vertical resolution of 140 nm and 2 nm, respectively. The recorded images were analyzed by the software Leica Map Premium 6.0 (Leica, Wetzlar, Germany) to evaluate the depth and the roughness factor. The textured surfaces were also characterized using a scanning electron microscope (ZEISS Supra 40VP, Jena, Germany) at an operating voltage of 8.0 kV, after coating the samples with a 30-nm thick gold layer.

### 2.5. Wettability Characterization

The static water contact angle (CA) of the laser-treated surfaces were recorded with a contact angle system (Krüss DSA 100 S, Hamburg, Germany). The system automatically controlled the deposition of 2 µL droplets of deionized water onto the samples and calculated the CA using the tangent drop profile fitting method. The ambient temperature was 22 °C during all the measurements.

### 2.6. Optical Characterization

To measure the intensity of the diffracted light by the structured glass samples, the setup shown schematically (top view) in Figure 1b was used. White light emitted by a tungsten lamp is directed to the sample holder by an optical fiber and focused on the sample by a bi-convex lens. Then, the transmitted light in the plane of the movement of the goniometer (angular resolution of 2°) is collected by a plano-convex lens and coupled to an optical fiber, which directs the light toward a UV-vis-NIR spectrometer (OceanOptics GmbH, HR2000+, Ostfildern, Germany). The measured intensities were normalized to the intensity corresponding to the incident light coming from the fiber. 

## 3. Results

### 3.1. Direct Fabrication of Microstructures on Glass

**Figure 3:**
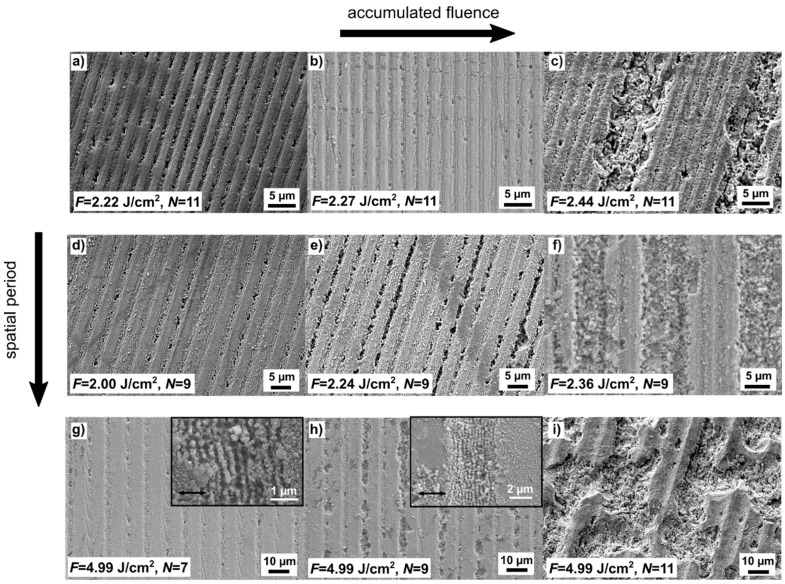
SEM images (secondary electron detector) of glass samples structured with line-like patterns and different spatial periods: (**a**–**c**) 2.3 µm, (**d**–**f**) 3.9 µm, and (**g**–**i**) 9.0 µm. The accumulated fluence dose increases from left to right. The inset of figures (**g**,**h**) show low spatial frequency LIPSS aligned perpendicularly to the laser polarization direction (arrows).

In order to verify the possibility to induce non-linear absorption mechanisms, a 5-mm thick soda lime glass has been irradiated with green (532 nm) laser radiation, employing a pulsed laser system (12 ps) with pulse energy *E*_p_ of at least 615 µJ and a compact optical setup for generating interference with spatial periods between 2.3 µm and 9.0 µm (see Figure 1). In a preliminary experiment, the laser fluence and spatial period were varied between 1.77 and 5.14 J/cm^2^ and 2.3 and 9.0 µm, respectively, to determine the influence of these parameters on the resulting topography. The selected SEM images of Figure 3 illustrate the influence of the accumulated fluence dose on the samples patterned by two-beam DLIP with spatial periods of 2.3 µm (Figure 3a–c), 3.9 µm (Figure 3d–f), and 9.0 µm (Figure 3g–i). The number of applied pulses *N* and fluence *F* are also shown on the images. In the first row of Figure 3, it can be seen that, as *F* increased from 2.22 to 2.44 J/cm^2^ (left to right), at a constant pulse number *N* = 11, the topography changed from a uniform texture with well-defined periodic grooves to a surface with severely damaged zones. A similar topography change was observed in the second row (Figure 3d–f), where, in this case, *F* was increased from 2.00 to 2.36 J/cm^2^, while *N* was held at 9. Particularly, for the lowest fluence (Figure 3d), the trenches are relatively shallow and their depth presents a modulation across the grooves direction, which may be attributed to a non-homogeneous ablation due to the Gaussian profile of the laser radiation. As the fluence increased to *F* = 2.24 J/cm^2^ (Figure 3e), the trenches became deeper but still a depth modulation can be observed. In the third row (Figure 3g–i), the fluence was kept constant at *F* = 4.99 J/cm^2^, and *N* was increased from 7 to 11. In this case, the trend is also repeated, i.e., as the accumulated fluence dose increased, the grooves’ depth increased as well and surface damage became more apparent. In the extreme case shown in Figure 3i, the surface was highly damaged, making the DLIP features hard to identify. The high-magnification images shown in the insets of Figure 3g,h allow the identification of ordered nanostructures within the micro-trenches, which are aligned perpendicularly to the radiation polarization direction (indicated with the arrows in the insets) and exhibit a spatial period of approximately ~300 nm. In agreement with previous works, the observed LIPSS spatial period is close to the ratio λ/n = 350 nm [55], where *n* = 1.52 is the refractive index of soda lime at a laser wavelength λ = 532 nm [66]. Considering their alignment and lateral feature size, these nanostructures can be identified as low spatial frequency LIPSS [67,68]. Moreover, the appearance of such LIPSS on the glass samples suggests that the absorption mechanism is mainly dictated by multi-photon absorption, as observed elsewhere [54,69,70,71].

Although DLIP ablation of transparent materials has not been extensively investigated so far, several works have modeled and discussed the multi-photon absorption processes in transparent materials, assuming a single incident beam. Particularly, Sun et al. have modeled ablation in soda lime glass considering the ultrafast dynamics of free-electrons using a rate equation for free-electron density including multi-photon ionization, avalanche ionization, and loss terms [72]. This model predicts that using pulse durations in the picosecond regime and laser fluences in the 1.7–5 J/cm^2^ range, as employed in our work, the process lies in the multi-photon ablation regime, in accordance with the results presented in this section.

**Figure 4:**
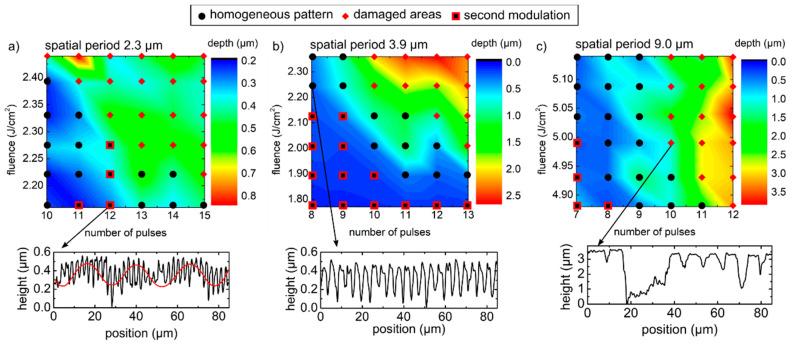
Depth diagrams of the line-like patterned samples with spatial periods of (**a**) 2.3 µm, (**b**) 3.9 µm, and (**c**) 9.0 µm, together with a representative extracted profile showing surfaces with (**a**) second modulation, (**b**) good overall uniformity and quality, and (**c**) significantly damaged areas.

Further information can be retrieved through a topography analysis by confocal microscopy. Varying the laser fluence and the pulse overlap (expressed as an equivalent number of pulses per laser spot) three contour plots (Figure 4) corresponding to samples structured with the spatial periods of 2.3 µm (a), 3.9 µm (b), and 9.0 µm (c) have been produced, summarizing the variation of the structure depth together with a qualitative interpretation of the surface quality. Namely, overlapped to the contour plots, different symbols are placed on the process variables that correspond to the actual samples, representing surfaces with either overall high homogeneity and quality (black circles) with significantly damaged areas (red diamonds) or showing an additional depth modulation due to the low intensity at the tails of the Gaussian beam (black/red squares), which are also known as a second modulation [73]. The general trend observed in the depth diagrams of Figure 4 is that, when the accumulated fluence is low, the microstructures are relatively shallow and a second depth modulation is noticed, as shown exemplarily in the profile of Figure 4a (the red line serves as a guide to the eye). As the accumulated fluence increased, the structures were deeper (i.e., up to 2.2 µm) and the topography homogeneity improved, as shown in the profile of Figure 4b. Finally, when the accumulated fluence dose was high enough, the surface became so damaged, that the DLIP texture lost its regularity (see the profile in Figure 4c). The maximum structure depths of those textures with a satisfactory texture quality were 0.53 µm, 1.12 µm, and 2.2 µm, for the patterns with spatial periods of 2.3 µm, 3.9 µm, and 9.0 µm, respectively, yielding maximum aspect ratios (depth to period ratio) of 0.23, 0.29, and 0.24, respectively. These diagrams also reveal that the process window for patterning glass with DLIP via non-linear absorption is very narrow and finding the optimum set of parameters can be challenging. As mentioned in Section 1, since the onset of non-linear absorption mechanisms strictly depends on the laser fluence dose, slight variations in pulse energy or pulse overlap may lead to large differences in the texturing results.

### 3.2. Wettability of Textured Glass Surfaces

The wettability of the laser-treated glass surfaces was characterized by measuring the static water contact angle (CA) using deionized water (2 µL of drop volume). The CA of the flat reference was 52°, indicating a moderate hydrophilic character of the used soda lime glass substrate. After the laser texturing, all the patterned surfaces showed a strong hydrophilic behavior, and some samples reached even a super-hydrophilic state, characterized by a CA lower than 5°, according to the definition by Drelich and coworkers [74].

**Figure 5:**
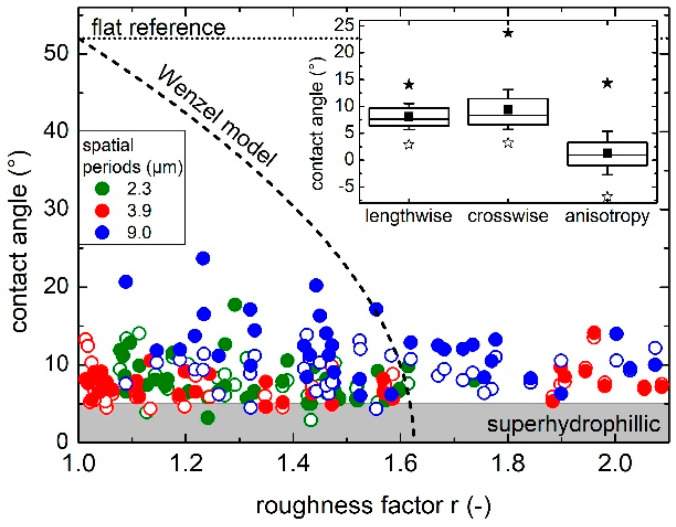
Water contact angle (CA) as a function of the roughness factor of all the line-like patterns. Open and filled symbols represent CA measured lengthwise and crosswise, respectively, to the direction of the grooves. The Wenzel model (dashed line) calculated with an equilibrium contact angle of 52° measured on the flat glass (dotted line) cannot explain the super-hydrophilic state reached for those samples with roughness factors below 1.6. The inset shows the statistical distribution of the CA measured lengthwise and crosswise as well as their difference (anisotropy). See text for details.

The inset in Figure 5 shows a box chart with the statistical distribution of the CA measured along and across the direction of the grooves (lengthwise and crosswise, respectively) of all structured samples, i.e., 108 patterned fields. The top, middle, and bottom lines of the boxes represent the 75%, 50% (or median), and 25% percentiles, respectively, whereas the filled squares indicate the mean value and the filled (empty) stars are the maximum (minimum) values. Both CAs showed similar mean values and statistical distribution, even though the standard deviation (whiskers) of the crosswise CA was 35% higher than the lengthwise measurement. A measure of the wetting anisotropy is also indicated in the chart, which was calculated as the difference between the lengthwise and crosswise CA. Analyzing this value, it can be observed that the mean value of the CA difference was only 1.3° and, therefore, it can be concluded that, despite the well-defined orientation of the DLIP grooves, the hydrophilic or super-hydrophilic states of the laser-treated samples were mainly isotropic. This behavior can be explained by taking into account the balance between capillary forces, surface tension, and gravity. For the textured glass surface, it can be assumed that the adhesion forces to the rough hydrophilic surface dominate and the droplet spread takes place in all directions regardless of the texture orientation [75,76,77].

In Figure 5, the CA of all the samples are plotted as a function of the samples’ roughness factor *r*, which is defined as the ratio of the real surface area to the projected surface area [78]. The open (filled) symbols stand for the CA measured lengthwise (crosswise) to the direction of the grooves. Strikingly, CAs close to 5° in both measuring directions were reached for roughness factors as low as 1.05. However, no clear trend was observed for the dependence of CA measured parallel or perpendicular to the DLIP grooves as the roughness factor increases.

The well-known Wenzel model can be used to explain the highly hydrophilic wetting state upon surface texturing [79]. According to this model, the liquid penetrates all the protrusions of the rough surface, wetting the entire surface of the solid. Then, the dependence of the contact angle θ_W_ as a function of the roughness factor *r* is given by *cos*(θ_W_) = *r* × *cos*(θ_0_), where θ_0_ is the CA of the flat surface [80]. As a result of the model, the hydrophilic behavior of an initially hydrophilic flat surface increases when its roughness increases. Although many studies suggest that this model fails to accurately predict the contact angle in real structured surfaces, it usually succeeds in providing a qualitative estimate of the general CA behavior [81,82,83,84]. Figure 5 also shows that the Wenzel model (dashed curve) assuming θ_0_ = 52° predicts a super-hydrophilic state if the roughness factor is higher than ~1.6. Nevertheless, in many structured samples, the super-hydrophilic state was reached for roughness factors well below that value, even approaching a roughness factor of 1. A possible explanation for this discrepancy may come from the fact that all the patterned samples have a hierarchical texture with several types of nano-scaled features that add to the DLIP trenches (Figure 3). These nanostructures, such as low and high spatial frequency LIPSS as well as defects arising from the redeposited material, chipping and non-uniform ablation, cannot be resolved by the used confocal microscope. Therefore, the actual roughness factor could be significantly higher (i.e., >1.6) than the measured values, correlating to a super-hydrophilic state according to Wenzel’s model. Superhydrophilicity on nanostructured and micro-structured glass with low anisotropy was also reported in many works for different types of geometrical textures and aspect ratios [12,20,85,86].

On the other hand, most of the measured values do not reach contact angles lower than 5° even though the samples show high roughness values. This behavior can be ascribed to the presence of atmospheric organic contaminants on the samples’ surface, which deposit on the surface through physisorption (Van der Waals bonds) [87]. In fact, a common source of such accidental contamination are hydrocarbons present in ambient air and this has been demonstrated to be present even in high-end nanofabrication clean rooms [88,89]. This contamination, which is very common in metal surfaces, has been confirmed on inert surfaces as SiO_2_ by means of spectroscopic investigations [90]. As commonly known, this thin organic layer leads to a decrease of the surface energy of the substrate and increases its hydrophobicity (i.e., an increase of the CA) [87], which may prevent the contact angles reported in this work to reach a value close to zero.

### 3.3. Optical Properties

**Figure 6:**
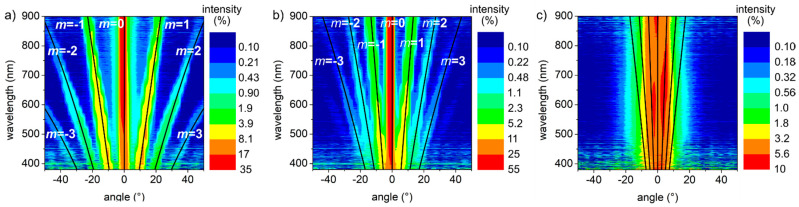
Spectral and angular dependence of the transmitted intensity through three selected structured samples with spatial periods of (**a**) 2.3 µm, (**b**) 3.9 µm, and (**c**) 9.0 µm. The black lines represent the diffraction grating equation for different diffraction orders *m*.

Using an in-house developed optical characterization setup (see Section 2 for details), the angular and spectral dependent intensity of the diffracted light in transmission mode was measured for all the line-like textures. Figure 6 shows the measured intensity (colors) for three selected samples with different spatial periods: (a) 2.3 µm (*F* = 2.27 J/cm^2^, *N* = 13), (b) 3.9 µm (*F* = 2.12 J/cm^2^, *N* = 9), and (c) 9.0 µm (*F* = 5.04 J/cm^2^, *N* = 11). The black lines represent the well-known diffraction grating equation [91] assuming the corresponding grating period and different diffraction orders *m*, namely *m* = 0, ±1, ±2, ±3. The intensity scale bar, on a logarithmic scale, is expressed as the percentage of the light coming from the optical fiber. As can be seen in Figure 6a,b, there is a very good match between the predicted diffraction orders using the grating equation with the measured intensities, confirming that the produced line-like textures behave effectively as relief diffraction gratings. However, for the sample with a spatial period of 9.0 µm (Figure 6c), the diffraction orders are tightly distributed and they cannot be separated from scattering effects, especially for short wavelengths. Consequently, the diagram presents a blur vertical band around the zero order instead of well-defined diffraction modes. The spatial period of the LIPSS (~300 nm) is shorter than the wavelengths used in this setup (380–900 nm), ruling out the propagation of higher diffraction [91]. Therefore, all the diffraction modes seen in Figure 6 are associated with the periodic DLIP microstructures.

**Figure 7:**
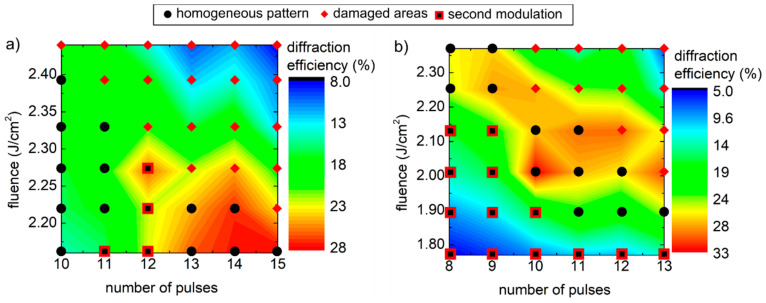
Diffraction efficiency determined for the line-like textures with spatial periods of (**a**) 2.3 µm and (**b**) 3.9 µm as a function of the process parameters.

Next, the diffraction efficiency *DE* is defined as the ratio between the sum of intensities of the diffracted orders (*m* = ±1, ±2, ±3) to the intensity of the incoming light from the optical fiber integrated in the vis-NIR spectral range, i.e., 380–900 nm. In Figure 7, the diffraction efficiency as a function of the number of laser pulses and fluence is shown for the line-like patterns with spatial periods of (a) 2.3 µm and (b) 3.9 µm. The same symbols displayed in the depth diagrams of Figure 4 representing the surface quality are overlapped with the diffraction efficiency data in Figure 7. It can be seen that the textures with both periods showed maximum diffraction efficiencies of approximately 30%. Since the maximum achievable *DE* depends strongly on the structure height and on the texture uniformity, the regions where the maximum *DE* in the diagrams of Figure 7 were found can be directly correlated with their topographical characteristics shown in Figure 4. For instance, the maximum *DE* for the textures with a spatial period of 2.3 µm were observed for low fluences and a high number of pulses that correspond to damage-free textures with relatively large structured depths of approximately ~0.5 µm (Figure 4). For a lower number of pulses, the structure depth and the diffraction efficiency became lower. As expected, in the regions with high accumulated fluence, i.e., high number of pulses with high fluence, which present a significant surface damage, the diffraction efficiency is low. A similar correlation between topography and *DE* was observed for the textures with a spatial period of 3.9 µm (Figure 7b), where a high diffraction efficiency region is located along the diagonal of the diagram, corresponding to the samples with a lower amount of defects. The maximum *DE* of 33% was found for homogeneous patterns with a structure depth of ~0.5 µm, but also *DE* values > 25% were found for structure depths around 1–1.3 µm. As indicated in Figure 4b and Figure 7b, second modulation effects were observed in several samples, reducing the overall surface uniformity. This phenomenon was particularly evident for low accumulated fluences (low number of pulses with low fluence) that led to low structure depths and low *DE*. These differences in the dependence of the chosen process parameters with the topography characteristics explain the different locations of the regions where the *DE* is maximized in Figure 7a,b.

Next, the maximum DE achieved in this study of 33% by the texture with a structure depth of 0.5 µm and spatial period of 3.9 µm is compared to an ideal thin sinusoidal phase grating, according to the Fraunhofer diffraction theory [92]. For the calculations, a dispersive index of refraction was considered for the soda lime glass [66]. It was assumed that the shape of the grating was a perfect sinusoidal function with a constant peak-to-valley height of 0.5 µm and the total diffraction efficiency was calculated as the sum of the efficiency of the first three orders averaged in the wavelength range of 380–900 nm. Considering that a portion of the incoming light is reflected at the air/glass interfaces, the calculation gave a diffraction efficiency of 57.8% (see Appendix A for details). The difference between the theoretical and experimental *DE* might come mainly from the deviation of the experimental topography from an ideal sinusoidal function. For instance, the depth of the texture is not uniform, and the presence of LIPSS and debris promote the scattering of incoming light into random directions. However, debris and other imperfections might be controlled by further optimization of the process. LIPSS formation is an intrinsic mechanism of the interaction between USP and the material and cannot be avoided.

In Table A1 of Appendix B, the samples with the maximum diffraction efficiencies and their process parameters are listed. In the table, the sample with a spatial period of 5.9 µm that had the deepest microstructure with satisfactory overall uniformity is also included.

### 3.4. Outlook on Four-Beam DLIP Structuring

**Figure 8:**
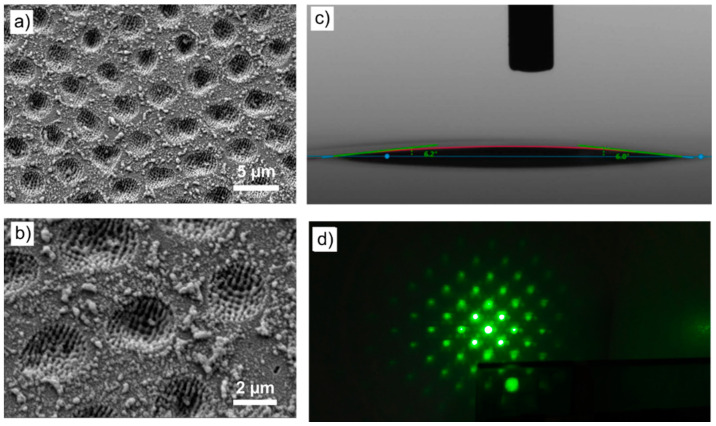
(**a**,**b**) SEM micrographs showing a glass surface structured by four-beam DLIP (5 applied pulses per spot at a fluence of 3.90 J/cm^2^). Aside from the well-defined periodic dot-like array (spatial period of 4.7 µm), LIPSS are visible inside the craters. (**c**) Photograph of a water droplet spreading on the textured surface with a contact angle of ~6°. (**d**) Diffraction pattern on a screen upon illuminating the structured glass with a green diode laser.

As stated above, many different texture geometries can be achieved by modifying the DLIP configuration such as by using a different number of laser beams, and changing their geometrical arrangement, polarization state, or scanning strategy. As a proof of concept, four-beam DLIP was used to generate a square array of holes with a spatial period of 4.7 µm by irradiating the glass surface with *N* = 5 pulses per spot with a pulse duration of 70 ps at a fluence of 3.90 J/cm^2^, as shown in the SEM images of Figure 8. LIPSS were also visible in the holes as well as a large amount of debris inside and around the holes produced by redeposited material and chipping. Confocal microscopy characterization revealed an average structure depth of 0.65 µm, representing an aspect ratio of 0.14.

The dot-like texture showed enhanced hydrophilicity, allowing water droplets to spread with contact angles of 6° (Figure 8c). Likewise, the textured glass acted as a relief diffraction grating when illuminated with a coherent light source. Figure 8d shows the two-dimensional diffraction pattern projected on a screen when the sample was illuminated with a green laser diode.

## 4. Conclusions

In this work, the feasibility of producing micropatterns with one and two dimensional periodicities on soda lime glass by non-linear absorption of interfering laser beams is demonstrated. The process window for obtaining homogenous line-like patterns without a significant damaged area turned to be particularly narrow and strongly dependent on the chosen spatial period. Uniform line-like textures with spatial periods of 2.3 µm, 3.9 µm, and 9.0 µm and maximum aspect ratios of 0.23, 0.29, and 0.24, respectively, were fabricated. The textures also presented low spatial frequency LIPSS with a periodicity of ~300 nm and aligned perpendicularly to the laser polarization. This effect also confirms the multi-photon absorption in the transparent soda lime glass.

The laser-treated surfaces showed enhanced functional properties. On the one hand, all the samples increased their hydrophilic characteristic, even reaching the super-hydrophilic state (CA < 5°) in some cases. On the other hand, homogenous structured samples with spatial periods of 2.3 µm and 3.9 µm diffracted white light into well-defined diffraction modes with efficiencies close to 30%. In this way, both enhanced properties can be combined in a single pattern to obtain multifunctional glass surfaces. As an example, patterning a line-like texture with a period of 2.3 µm with 14 pulses at a fluence of 2.22 J/cm^2^ resulted in a super-hydrophilic surface able to diffract light with an efficiency of 25%. 

It was also demonstrated the feasibility of employing four-beam DLIP to pattern periodic dot-like arrays with ultra-short laser pulses with a wavelength in the visible spectrum. Further investigations need to be done to optimize the process and to study the modified surface properties.

Although the throughputs achieved in this study (~3 cm^2^/min) are relatively low to be attractive for industrial applications, the DLIP method can be scaled up to mass production, e.g., throughputs ~1 m^2^/min, by using laser sources with higher power output that allows larger spot sizes, by optimizing the DLIP optics and employing faster beam guidance systems, such as galvo-scanners or polygon scanners.

## Figures and Tables

**Table 1:** Process parameters for structuring line-like and dot-like textures.

Parameter	Line-Like Textures			Dot-Like Textures	
Spatial period [µm]	2.3	3.9	9.0	2.3	4.7
Spot size [µm]	74	52	54	45	
Hatch [µm]	23.0	15.6	18.0	16.5	13.4
Fluence [J/cm^2^]	2.16–2.44	1.77–2.36	4.88–5.14	3.77–3.90	2.67–3.71
Number of pulses	10–15	8–13	7–12	5–7	2–6
Repetition rate [kHz]	10			1	

## Data Availability

The data presented in this study are available on request from the corresponding author.

## Appendix A. Calculation of Diffraction Efficiency of a Thin Sinusoidal Phase Grating

Following the Fraunhofer diffraction calculations presented in Reference [92], the diffraction efficiency of the diffraction mode *m* of an ideal thin sinusoidal phase grating can be estimated as:

(A1) $$\eta_{m}=J_{m}^{2}\left(\beta /2\right),$$

where *J* is the Bessel function of the first kind and *β* is the maximum phase contrast between the waves emerging at the back side of the grating and is given by:

(A2) $$\beta \;=\;\frac{2\pi h}{\lambda }\left(n\left(\lambda \right)-1\right),$$

where *n*(*λ*) is the dispersive refractive index of soda lime glass and *h* is the grating depth. The diffraction efficiency considering the orders *m* = ±1, ±2 and ±3 is:

(A3) $$\eta \;=\;2\times \left(J_{1}^{2}+J_{2}^{2}+J_{3}^{2}\right),$$

which is also a function of the wavelength. The reflection at the glass/air interface can be calculated by Reference [91].

(A4) $$R\left(\lambda \right)\;=\;{\left(\frac{n-1}{n+1}\right)}^{2}.$$

Considering that the light interacts with the air/glass interface of the front and back sides of the grating, the transmitted intensity is reduced due to reflection by a factor (1 − *R*)^2^.

Finally, the averaged diffraction efficiency across the visible-NIR spectrum is calculated as:

$$DE\;=\;\frac{1}{900\mathrm{nm}-380\mathrm{nm}}\int_{380\mathrm{nm}}^{900\mathrm{nm}}{\left(1-R\left(\lambda \right)\right)}^{2}\eta \left(\lambda \right)d\lambda .$$

## Appendix B

**Table A1:** Selected samples with their spatial periods, process parameters, structure depth, diffraction efficiency, and contact angle. The samples with the spatial periods of 2.3 µm and 3.9 µm were selected because they have shown the maximum diffraction efficiency for each period. In turn, the sample with the spatial period of 5.9 µm was selected, as it had the deepest microstructures with satisfactory homogeneity.

Spatial period [µm]	2.3	3.9	5.9
Fluence [J/cm2]	2.16	2.01	4.88
Number of pulses	14	10	11
Structure depth [µm]	0.34	0.30	2.21
Diffraction efficiency [%]	27.8	32.7	-
Contact angle [°]	7.2	5.9	10.1

## References

[1] Righini G.C., Chiappini A. (2014). Glass Optical Waveguides: A Review of Fabrication Techniques. Opt. Eng..

[2] Zhang H., Lee Y.Y., Leck K.J., Kim N.Y., Ying J.Y. (2007). Recyclable Hydrophilic- Hydrophobic Micropatterns on Glass for Microarray Applications. Langmuir.

[3] Ainslie K.M., Desai T.A. (2008). Microfabricated Implants for Applications in Therapeutic Delivery, Tissue Engineering, and Biosensing. Lab Chip.

[4] Bertin A., Schlaad H. (2009). Mild and Versatile (Bio-)Functionalization of Glass Surfaces via Thiol−Ene Photochemistry. Chem. Mater..

[5] Ottevaere H., Cox R., Herzig H.P., Miyashita T., Naessens K., Taghizadeh M., Völkel R., Woo H.J., Thienpont H. (2006). Comparing Glass and Plastic Refractive Microlenses Fabricated with Different Technologies. J. Opt. A Pure Appl. Opt..

[6] Kuna L., Haase A., Sommer C., Zinterl E., Krenn J.R., Wenzl F.P., Pachler P., Hartmann P., Tasch S., Leising G. (2008). Improvement of Light Extraction from High-Power Flip-Chip Light-Emitting Diodes by Femtosecond Laser Direct Structuring of the Sapphire Backside Surface. J. Appl. Phys..

[7] Fuchs A., Kanoufi F., Combellas C., Shanahan M.E.R. (2007). Wetting and Surface Properties of (Modified) Fluoro-Silanised Glass. Colloid Surf. A.

[8] Kolli M., Hamidouche M., Bouaouadja N., Fantozzi G. (2009). HF Etching Effect on Sandblasted Soda-Lime Glass Properties. J. Eur. Ceram. Soc..

[9] Wei M., Bowman R.S., Wilson J.L., Morrow N.R. (1993). Wetting Properties and Stability of Silane-Treated Glass Exposed to Water, Air, and Oil. J. Colloid Interf. Sci..

[10] Fang Z., Qiu X., Qiu Y., Kuffel E. (2006). Dielectric Barrier Discharge in Atmospheric Air for Glass-Surface Treatment to Enhance Hydrophobicity. IEEE Trans. Plasma Sci..

[11] Kim S.H., Yang Y., Kim M., Nam S.-W., Lee K.-M., Lee N.Y., Kim Y.S., Park S. (2007). Simple Route to Hydrophilic Microfluidic Chip Fabrication Using an Ultraviolet (UV)-Cured Polymer. Adv. Funct. Mater..

[12] Domke M., Sonderegger G., Kostal E., Matylitsky V., Stroj S. (2019). Transparent Laser-Structured Glasses with Superhydrophilic Properties for Anti-Fogging Applications. Appl. Phys. A.

[13] Chen Y., Zhang Y., Shi L., Li J., Xin Y., Yang T., Guo Z. (2012). Transparent Superhydrophobic/Superhydrophilic Coatings for Self-Cleaning and Anti-Fogging. Appl. Phys. Lett..

[14] Akhtar N., Thomas P.J., Svardal B., Almenningen S., de Jong E., Magnussen S., Onck P.R., Fernø M.A., Holst B. (2018). Pillars or Pancakes? Self-Cleaning Surfaces without Coating. Nano Lett..

[15] Xue Z., Wang S., Lin L., Chen L., Liu M., Feng L., Jiang L. (2011). A Novel Superhydrophilic and Underwater Superoleophobic Hydrogel-Coated Mesh for Oil/Water Separation. Adv. Mater..

[16] Zhang S., Jiang G., Gao S., Jin H., Zhu Y., Zhang F., Jin J. (2018). Cupric Phosphate Nanosheets-Wrapped Inorganic Membranes with Superhydrophilic and Outstanding Anticrude Oil-Fouling Property for Oil/Water Separation. ACS Nano.

[17] Zhang W., Bunte E., Worbs J., Siekmann H., Kirchhoff J., Gordijn A., Hüpkes J. (2010). Rough Glass by 3d Texture Transfer for Silicon Thin Film Solar Cells. Phys. Status Solidi C.

[18] Chen T.-G., Yu P., Tsai Y.-L., Shen C.-H., Shieh J.-M., Tsai M.-A., Kuo H.-C. (2012). Nano-Patterned Glass Superstrates with Different Aspect Ratios for Enhanced Light Harvesting in a-Si: H Thin Film Solar Cells. Opt. Express.

[19] Yang G., van Swaaij R.A., Tan H., Isabella O., Zeman M. (2015). Modulated Surface Textured Glass as Substrate for High Efficiency Microcrystalline Silicon Solar Cells. Sol. Energy Mater. Sol. Cells.

[20] Son J., Kundu S., Verma L.K., Sakhuja M., Danner A.J., Bhatia C.S., Yang H. (2012). A Practical Superhydrophilic Self Cleaning and Antireflective Surface for Outdoor Photovoltaic Applications. Sol. Energy Mater. Sol. Cells.

[21] Verma L.K., Sakhuja M., Son J., Danner A.J., Yang H., Zeng H.C., Bhatia C.S. (2011). Self-Cleaning and Antireflective Packaging Glass for Solar Modules. Renew. Energy.

[22] Trinh Q.H., Hossain M.d.M., Kim S.H., Mok Y.S. (2018). Tailoring the Wettability of Glass Using a Double-Dielectric Barrier Discharge Reactor. Heliyon.

[23] Terpiłowski K., Rymuszka D., Goncharuk O.V., Sulym I.Y., Gun’ko V.M. (2015). Wettability of Modified Silica Layers Deposited on Glass Support Activated by Plasma. Appl. Surf. Sci..

[24] Kontziampasis D., Boulousis G., Smyrnakis A., Ellinas K., Tserepi A., Gogolides E. (2014). Biomimetic, Antireflective, Superhydrophobic and Oleophobic PMMA and PMMA-Coated Glass Surfaces Fabricated by Plasma Processing. Microelectron. Eng..

[25] Wang S., Deng Y., Yang L., Shi X., Yang W., Chen Z.-G. (2018). Enhanced Antibacterial Property and Osteo-Differentiation Activity on Plasma Treated Porous Polyetheretherketone with Hierarchical Micro/Nano-Topography. J. Biomater. Sci. Polym. Ed..

[26] Kontziampasis D., Trantidou T., Regoutz A., Humphrey E.J., Carta D., Terracciano C.M., Prodromakis T. (2016). Effects of Ar and O2 Plasma Etching on Parylene C: Topography versus Surface Chemistry and the Impact on Cell Viability. Plasma Process. Polym..

[27] Awaja F., Tripathi M., Wong T.-T., O’Brien T., Speranza G. (2018). The Chemistry and Topography of Stabilized and Functionalized Graphene Oxide Coatings. Plasma Process Polym..

[28] Hein E., Fox D., Fouckhardt H. (2011). Glass Surface Modification by Lithography-Free Reactive Ion Etching in an Ar/CF4-Plasma for Controlled Diffuse Optical Scattering. Surf. Coat. Tech..

[29] Jain H., Vlcek M. (2008). Glasses for Lithography. J. Non Cryst. Solids.

[30] Hicks E.M., Lyandres O., Hall W.P., Zou S., Glucksberg M.R., Van Duyne R.P. (2007). Plasmonic Properties of Anchored Nanoparticles Fabricated by Reactive Ion Etching and Nanosphere Lithography. J. Phys. Chem. C.

[31] Hannes B., Vieillard J., Chakra E.B., Mazurczyk R., Mansfield C.D., Potempa J., Krawczyk S., Cabrera M. (2008). The Etching of Glass Patterned by Microcontact Printing with Application to Microfluidics and Electrophoresis. Sensors Actuators B Chem..

[32] Coltro W.K.T., Piccin E., da Silva J.A.F., do Lago C.L., Carrilho E. (2007). A Toner-Mediated Lithographic Technology for Rapid Prototyping of Glass Microchannels. Lab Chip.

[33] Garcia R., Knoll A.W., Riedo E. (2014). Advanced Scanning Probe Lithography. Nat. Nanotechnol..

[34] Lim M.P., Guo X., Grunblatt E.L., Clifton G.M., Gonzalez A.N., LaFratta C.N. (2018). Augmenting Mask-Based Lithography with Direct Laser Writing to Increase Resolution and Speed. Opt. Express.

[35] Buividas R., Mikutis M., Juodkazis S. (2014). Surface and Bulk Structuring of Materials by Ripples with Long and Short Laser Pulses: Recent Advances. Prog. Quantum Electron..

[36] Cheng J., Liu C., Shang S., Liu D., Perrie W., Dearden G., Watkins K. (2013). A Review of Ultrafast Laser Materials Micromachining. Opt. Laser Technol..

[37] Lorenz P., Ehrhardt M., Zimmer K. (2012). Laser-Induced Front Side and Back Side Etching of Fused Silica with KrF and XeF Excimer Lasers Using Metallic Absorber Layers: A Comparison. Appl. Surf. Sci..

[38] Zhang J., Sugioka K., Midorikawa K. (1998). Laser-Induced Plasma-Assisted Ablation of Fused Quartz Using the Fourth Harmonic of a Nd+: YAG Laser. Appl. Phys. A.

[39] Shkuratova V.A., Kostyuk G.K., Sergeev M.M., Zakoldaev R.A., Yakovlev E.B. (2019). Speckle-Free Smoothing of Coherence Laser Beams by a Homogenizer on Uniaxial High Birefringent Crystal. Opt. Mater. Express.

[40] Alamri S., Sürmann P.A., Lasagni A.F., Kunze T. (2020). Interference-Based Laser-Induced Micro-Plasma Ablation of Glass. Adv. Opt. Tech..

[41] Niino H., Kawaguchi Y., Sato T., Narazaki A., Ding X., Kurosaki R. (2004). Surface Microfabrication of Fused Silica Glass by UV Laser Irradiation. Proceedings of the Photon Processing in Microelectronics and Photonics III.

[42] Herman P.R., Marjoribanks R.S., Oettl A., Chen K., Konovalov I., Ness S. (2000). Laser Shaping of Photonic Materials: Deep-Ultraviolet and Ultrafast Lasers. Appl. Surf. Sci..

[43] Schwerter M., Gräbner D., Hecht L., Vierheller A., Leester-Schädel M., Dietzel A. (2016). Surface-Passive Pressure Sensor by Femtosecond Laser Glass Structuring for Flip-Chip-in-Foil Integration. J. Microelectromech. Syst..

[44] Ihlemann J., Wolff-Rottke B. (1996). Excimer Laser Micro Machining of Inorganic Dielectrics. Appl. Surf. Sci..

[45] Stoian R., Boyle M., Thoss A., Rosenfeld A., Korn G., Hertel I.V., Campbell E.E.B. (2002). Laser Ablation of Dielectrics with Temporally Shaped Femtosecond Pulses. Appl. Phys. Lett..

[46] Du D., Liu X., Korn G., Squier J., Mourou G. (1994). Laser-induced Breakdown by Impact Ionization in SiO2 with Pulse Widths from 7 Ns to 150 Fs. Appl. Phys. Lett..

[47] Schaffer C.B., Brodeur A., Mazur E. (2001). Laser-Induced Breakdown and Damage in Bulk Transparent Materials Induced by Tightly Focused Femtosecond Laser Pulses. Meas. Sci. Technol..

[48] Mao S.S., Quéré F., Guizard S., Mao X., Russo R.E., Petite G., Martin P. (2004). Dynamics of Femtosecond Laser Interactions with Dielectrics. Appl. Phys. A.

[49] Jamshidi-Ghaleh K., Masalehdan H. (2009). Modeling of Nonlinear Responses in BK7 Glass under Irradiation of Femtosecond Laser Pulses. Opt. Quantum Electron..

[50] Sun M., Eppelt U., Russ S., Hartmann C., Siebert C., Zhu J., Schulz W. (2013). Numerical Analysis of Laser Ablation and Damage in Glass with Multiple Picosecond Laser Pulses. Opt. Express.

[51] Rohloff M., Das S.K., Höhm S., Grunwald R., Rosenfeld A., Krüger J., Bonse J. (2012). Formation of Laser-Induced Periodic Surface Structures on Fused Silica upon Multiple Parallel Polarized Double-Femtosecond-Laser-Pulse Irradiation Sequences. Appl. Surf. Sci..

[52] Ahsan M.d.S., Lee M.S., Hasan M.K., Noh Y.-C., Sohn I.-B., Ahmed F., Jun M.B.G. (2015). Formation Mechanism of Self-Organized Nano-Ripples on Quartz Surface Using Femtosecond Laser Pulses. Optik.

[53] Yin K., Wang C., Duan J., Guo C. (2016). Femtosecond Laser-Induced Periodic Surface Structural Formation on Sapphire with Nanolayered Gold Coating. Appl. Phys. A.

[54] Alamri S., Fraggelakis F., Kunze T., Krupop B., Mincuzzi G., Kling R., Lasagni A.F. (2019). On the Interplay of DLIP and LIPSS Upon Ultra-Short Laser Pulse Irradiation. Materials.

[55] Gräf S., Kunz C., Müller F.A. (2017). Formation and Properties of Laser-Induced Periodic Surface Structures on Different Glasses. Materials.

[56] Gnilitskyi I., Derrien T.J.-Y., Levy Y., Bulgakova N.M., Mocek T., Orazi L. (2017). High-Speed Manufacturing of Highly Regular Femtosecond Laser-Induced Periodic Surface Structures: Physical Origin of Regularity. Sci. Rep..

[57] Lasagni A.F. (2017). Laser Interference Patterning Methods: Possibilities for High-Throughput Fabrication of Periodic Surface Patterns. Adv. Opt. Tech..

[58] Indrišiūnas S., Voisiat B., Gedvilas M., Račiukaitis G. (2017). New Opportunities for Custom-Shape Patterning Using Polarization Control in Confocal Laser Beam Interference Setup. J. Laser. Appl..

[59] Lasagni A., Mücklich F. (2009). FEM Simulation of Periodical Local Heating Caused by Laser Interference Metallurgy. J. Mater. Proc. Technol..

[60] Gachot C., Catrin R., Lasagni A., Schmid U., Mücklich F. (2009). Comparative Study of Grain Sizes and Orientation in Microstructured Au, Pt and W Thin Films Designed by Laser Interference Metallurgy. Appl. Surf. Sci..

[61] Daniel C., Lasagni A., Mücklich F. (2004). Stress and Texture Evolution of Ni/Al Multi-Film by Laser Interference Irradiation. Surf. Coat. Technol..

[62] Kawamura K., Sarukura N., Hirano M., Hosono H. (2000). Holographic Encoding of Permanent Gratings Embedded in Diamond by Two Beam Interference of a Single Femtosecond Near-Infrared Laser Pulse. Jpn. J. Appl. Phys..

[63] Kawamura K., Ogawa T., Sarukura N., Hirano M., Hosono H. (2000). Fabrication of Surface Relief Gratings on Transparent Dielectric Materials by Two-Beam Holographic Method Using Infrared Femtosecond Laser Pulses. Appl. Phys. B.

[64] Hosono H., Kawamura K., Matsuishi S., Hirano M. (2002). Holographic Writing of Micro-Gratings and Nanostructures on Amorphous SiO2 by near Infrared Femtosecond Pulses. Nucl. Instrum. Methods B.

[65] Lasagni A.F., Roch T., Berger J., Kunze T., Lang V., Beyer E. To Use or Not to Use (Direct Laser Interference Patterning), That Is the Question. Proceedings of the SPIE 9351, Laser-based Micro- and Nanoprocessing IX.

[66] Rubin M. (1985). Optical Properties of Soda Lime Silica Glasses. Sol. Energy Mater..

[67] Bonse J., Höhm S., Kirner S.V., Rosenfeld A., Krüger J. (2017). Laser-Induced Periodic Surface Structures—A Scientific Evergreen. IEEE J. Sel. Top. Quantum Electron..

[68] Bonse J., Krüger J., Höhm S., Rosenfeld A. (2012). Femtosecond Laser-Induced Periodic Surface Structures. J. Laser Appl..

[69] Cheng G., Mishchik K., Mauclair C., Audouard E., Stoian R. (2009). Ultrafast Laser Photoinscription of Polarization Sensitive Devices in Bulk Silica Glass. Opt. Express.

[70] Mishchik K., Cheng G., Huo G., Burakov I.M., Mauclair C., Mermillod-Blondin A., Rosenfeld A., Ouerdane Y., Boukenter A., Parriaux O. (2010). Nanosize Structural Modifications with Polarization Functions in Ultrafast Laser Irradiated Bulk Fused Silica. Opt. Express.

[71] Soileau M. (1984). Ripple Structures Associated with Ordered Surface Defects in Dielectrics. IEEE J. Quantum Electron..

[72] Sun M., Eppelt U., Russ S., Hartmann C., Siebert C., Zhu J., Schulz W. (2012). Laser Ablation Mechanism of Transparent Dielectrics with Picosecond Laser Pulses. Proceedings of the Laser-Induced Damage in Optical Materials.

[73] Aguilar-Morales A.I., Alamri S., Kunze T., Lasagni A.F. (2018). Influence of Processing Parameters on Surface Texture Homogeneity Using Direct Laser Interference Patterning. Opt. Laser Technol..

[74] Drelich J., Chibowski E., Meng D.D., Terpilowski K. (2011). Hydrophilic and Superhydrophilic Surfaces and Materials. Soft Matter.

[75] Lee S.J., Ha N., Kim H. (2019). Superhydrophilic–Superhydrophobic Water Harvester Inspired by Wetting Property of Cactus Stem. ACS Sustain. Chem. Eng..

[76] Son H.H., Seo G.H., Jeong U., Kim S.J. (2017). Capillary Wicking Effect of a Cr-Sputtered Superhydrophilic Surface on Enhancement of Pool Boiling Critical Heat Flux. Int. J. Heat Mass Transf..

[77] Si Y., Dong Z., Jiang L. (2018). Bioinspired Designs of Superhydrophobic and Superhydrophilic Materials. ACS Cent. Sci..

[78] Ran C., Ding G., Liu W., Deng Y., Hou W. (2008). Wetting on Nanoporous Alumina Surface: Transition between Wenzel and Cassie States Controlled by Surface Structure. Langmuir.

[79] Wenzel R.N. (1936). Resistance of Solid Surfaces to Wetting by Water. Ind. Eng. Chem..

[80] Wolansky G., Marmur A. (1999). Apparent Contact Angles on Rough Surfaces: The Wenzel Equation Revisited. Colloid Surf. A.

[81] Joanny J.F., De Gennes P.-G. (1984). A Model for Contact Angle Hysteresis. J. Chem. Phys..

[82] Leroy F., Müller-Plathe F. (2011). Rationalization of the Behavior of Solid- Liquid Surface Free Energy of Water in Cassie and Wenzel Wetting States on Rugged Solid Surfaces at the Nanometer Scale. Langmuir.

[83] Marmur A., Bittoun E. (2009). When Wenzel and Cassie Are Right: Reconciling Local and Global Considerations. Langmuir.

[84] Erbil H.Y., Cansoy C.E. (2009). Range of Applicability of the Wenzel and Cassie- Baxter Equations for Superhydrophobic Surfaces. Langmuir.

[85] Yu E., Kim S.-C., Lee H.J., Oh K.H., Moon M.-W. (2015). Extreme Wettability of Nanostructured Glass Fabricated by Non-Lithographic, Anisotropic Etching. Sci. Rep..

[86] Kostal E., Stroj S., Kasemann S., Matylitsky V., Domke M. (2018). Fabrication of Biomimetic Fog-Collecting Superhydrophilic–Superhydrophobic Surface Micropatterns Using Femtosecond Lasers. Langmuir.

[87] Li Z., Wang Y., Kozbial A., Shenoy G., Zhou F., McGinley R., Ireland P., Morganstein B., Kunkel A., Surwade S.P. (2013). Effect of Airborne Contaminants on the Wettability of Supported Graphene and Graphite. Nat. Mater..

[88] Kurokawa A., Odaka K., Azuma Y., Fujimoto T., Kojima I. (2009). Diagnosis and Cleaning of Carbon Contamination on SiO2 Thin Film. J. Surf. Anal..

[89] Smith P.J., Lindley P.M. Analysis of Organic Contamination in Semiconductor Processing. Proceedings of the AIP Conference Proceedings.

[90] Shinozaki A., Arima K., Morita M., Kojima I., Azuma Y. (2003). FTIR-ATR Evaluation of Organic Contaminant Cleaning Methods for SiO2 Surfaces. Anal. Sci..

[91] Hecht E. (2001). Optics.

[92] Goodman J.W. (1996). Introduction to Fourier Optics.

